# The Effect Of Alpha‐Synuclein On The Expression Of Genes Encoded In Mitochondrial DNA In Human Astrocyte

**DOI:** 10.1002/alz70855_103262

**Published:** 2025-12-24

**Authors:** Büşra Şengül, Zuhal Yurttaş, Tugay Çamoğlu, Bilge Nur Bilge, Sümeyra Ildız, Nazlıcan İlhan, Erdinc Dursun, Duygu Gezen‐Ak

**Affiliations:** ^1^ Institute of Neurological Sciences, Department of Neuroimmunology, Istanbul University‐Cerrahpasa, Istanbul, Turkey; ^2^ Institute of Neurological Sciences, Department of Neuroscience, Brain and Neurodegenerative Disorders Research Laboratories, Istanbul University‐Cerrahpasa, Istanbul, Turkey; ^3^ Institute of Neurological Sciences, Department of Neuroscience, Brain and Neurodegenerative Disorders Research Laboratories, Istanbul University‐Cerrahpaşa, Istanbul, Turkey; ^4^ Cerrahpasa Faculty of Medicine, Department of Medical Biology, Istanbul University‐Cerrahpasa, Istanbul, Turkey; ^5^ Department of Neuroscience, Institute of Neurological Sciences, Istanbul University‐Cerrahpasa, Turkey, Istanbul, Turkey

## Abstract

**Background:**

Mitochondrial dysfunction in energy metabolism is considered one of the early features of neurodegenerative diseases (1). In Parkinson's disease (PD), mitochondrial functions are among the earliest disrupted neurodegeneration pathways (2). The translocation of alpha‐synuclein (α‐syn), encoded by the SNCA gene, the major protein of Lewy bodies seen in PD, to mitochondria has been demonstrated (3). Although the importance of α‐syn in PD pathology is known, its specific roles within mitochondria must be better understood (4). In this study, we aimed to investigate the regulatory effects of SNCA gene overexpression on the expression of mitochondrial DNA (mtDNA) encoded genes.

**Method:**

Human astrocytes were transfected with a plasmid carrying the SNCA gene and SNCA was overexpressed. The group transfected with the MOCK plasmid was used as a control. RNA isolations were performed 24 and 48 hours after SNCA transfection. Following cDNA synthesis, the expression of 13 respiratory complex genes encoded by mtDNA, two mitochondrial rRNA genes, and three mitochondrial tRNA genes was investigated using qRT‐PCR. Statistical analysis of the results was performed using a one‐way ANOVA test with GraphPad Prism 8.

**Result:**

After 24 hours of SNCA transfection, the expression of MTND2 (* *p* <0.05) and MTtRNA3 (* *p* <0.05; ** *p* <0.01) increased in the SNCA overexpression group compared to both the MOCK group and the control group. After 48 hours of SNCA transfection, in the SNCA group, mRNA expression levels of MTCYB, MTCO3, MTtRNA1, and MTD‐loop were statistically significantly increased compared to the MOCK group (* *p* <0.05).

**Conclusion:**

These findings indicate that alpha‐synuclein overexpression can modulate the expression of mitochondrial genes, highlighting its potential role in mitochondrial dysfunction associated with Parkinson's disease. This study provides new insights into the molecular interactions between alpha‐synuclein and mitochondrial gene regulation, offering a basis for future investigations into its contribution to neurodegeneration in PD.

